# Draft Genome Sequence of Shiga Toxin-Producing *Escherichia coli* Strain D92/09

**DOI:** 10.1128/genomeA.00805-15

**Published:** 2015-07-30

**Authors:** Ana Carolina Da Silva Santos, Josias Rodrigues

**Affiliations:** Laboratory of Medical Bacteriology, Department of Microbiology and Immunology, Institute of Biosciences of the State University of São Paulo (IBB-UNESP), Botucatu SP, Brazil

## Abstract

*Escherichia coli* is suspected to be involved with Crohn’s disease. Adherence and invasion to epithelial cells are properties commonly observed in these bacteria. Here, we present a draft genome sequence of *E. coli* D92/09, a multidrug-resistant strain, which besides showing these properties produces Shiga cytotoxin-1 and possibly other toxins.

## GENOME ANNOUNCEMENT

We reported recently the detection, in a Crohn’s disease (CD) patient, of an *Escherichia coli* strain displaying virulence markers of three classical diarrheagenic pathotypes: enterohemorrhagic *E. coli* (EHEC) (*eae* and *stx1*), enteroaggregative *E. coli* (EAEC) (*aggR* and aggregative adherence to HEp-2 cells), and enteroinvasive *E. coli* (EIEC) (invasive ability into cultured eukaryotic cells) (1). The patient was an adult woman who underwent ileal resection 1 year earlier to repair an intestinal obstruction. The bacteria, which were detected both in ileal biopsy and stool samples from a patient, probably consisted of a dominant or a single *E. coli* clone, because all of 20 colonies picked from the MacConkey agar plates streaked with the clinical samples showed phenotypic (biochemical profile and interactions with cultured cells) and genetic (EcoR phylogroup, virulence gene profile and multilocus sequence type) similarity (1). One of the ileal mucosa isolates, designated strain D92/09, had its whole genome sequenced using the Ion Torrent Personal Genome Machine (PGM) platform. Bacterial DNA was extracted from LB overnight cultures with the QiAamp minikit (Qiagen, Hilden, Germany), and the genome library was prepared using 1 µg of DNA and an Ion Xpress Plus fragment library kit comprising the Ion Shear chemistry, according to the user’s guide. The resulting DNA fragments were separated in an E-gel electrophoresis system (Life Technologies, Guilford, CT), from which 400-bp fragments were recovered. The fragments were then ligated to Ion Sphere particles (ISP) for clonal amplification in an emulsion PCR carried out with the Ion OneTouch instrument (Life Technologies). After checking the quality of amplification, the ISP beads were loaded onto a 314 Chip, where the sequencing proceeded in the PGM.

Genome sequence features and sequencing quality parameters were available in files accessed in the Torrent Server by the Torrent browser software, which revealed a total of 399,241 reads comprising 16.5× genome coverage. *De novo* genome sequence assembly was carried out with the Assembler plugin, using as a reference the adherent-invasive *E. coli* strain LF82 genome (2), to which 76% of the D92/09 strain bases were aligned. This whole genome (WG) of the strain is composed of 5,079,894 bases distributed in 343 contigs, which were reordered with the Mauve aligner software (3) for annotation in the RAST server (4). The D92/09 WG has a G+C content of 50.47%. A total of 5,893 coding sequences (CDS), 18 rRNAs, and 66 tRNAs were identified. Virulence-related subsystem features consisted of 11 adhesion-associated factors, 103 sequences conferring resistance to antibiotics and toxic compounds, and 18 *Mycobacterium*- and *Listeria*-related elements necessary for cell invasion and intracellular persistence; in addition, 88 phage or prophage sequences were identified. The D92/09 WG showed 97% identity with recently described nitrofurantoin-resistant sequence type 2747 (ST2747) (5) and STEC O104:H4/2011C-3493 (NCBI reference sequence NC_018658.1) *E. coli* strains. A total of 145,211 bp corresponded to plasmid sequences, part of which matched pO111:H- (NCBI reference sequence NC_013370.1) and several antibiotic resistance factors, including the extended-spectrum β-lactamase (ESBL) gene carrying plasmid pH 2332 166 (NCBI reference sequence NC_025175.1).

### Nucleotide sequence accession numbers.

This whole-genome shotgun project has been deposited at DDBJ/EMBL/GenBank under the accession no. LEKZ00000000. The version described in this paper is version LEKZ01000000.
